# Comparing the association of cardiovascular nursing care with blood pressure and length of stay of in-patients with coronary artery disease in Wuhan, China

**DOI:** 10.4314/ahs.v20i4.23

**Published:** 2020-12

**Authors:** Fatina B Ramadhani, Yilan Liu, Xue Jing, Ye Qing, Han Xiong, Fengjian Zhang, Pian Wei

**Affiliations:** 1 Department of Nursing, Tongji Medical College, Huazhong University of Science and Technology; 2 Department of Nursing, Union Hospital of Tongji Medical College, Huazhong University of Science and Technology; 3 Department of cardiology, Union Hospital of Tongji Medical College, Huazhong University of science and Technology; 4 Department of Clinical Nursing, Muhimbili University of Health and Allied Sciences; 5 College of Arts and Science of Jianghan University

**Keywords:** Cardiovascular nursing care, blood pressure, in-patients, coronary artery, Wuhan, China

## Abstract

**Background:**

Coronary artery disease is a leading cause of morbidity and mortality worldwide. Comorbidity-like hypertension has been among the major risks of coronary artery disease. Recent evidence identified multiple benefits of cardiovascular nursing care to coronary patients. However, little has been appraised on benefits regarding patients' blood pressure control and length of hospitalisation.

**Objective:**

To compare the association of cardiovascular nursing care delivered to coronary artery patients with patients' blood pressure and length of stay.

**Methods:**

Records based retrospective design was applied at a large teaching hospital in Wuhan, China. SPSS 21 version was used for data entry and analysis with univariate and multivariate logistic regression models for comparing study variables.

**Results:**

Of 300 patients, 224 (74.7%) were known to be hypertensive and admitted with subnormal blood pressure. Cardiovascular nursing care like “assess to grade pain severity on 1–10 scale” and “counsel patient to cope with stress” were six and three times more likely to contribute improved patients' blood pressure (AOR=5.8; 95%CI: 2.8–12.2, p=0.001) and (AOR=3.1; 95%CI: 1.2–7.8, p=0.015) respectively. No significant difference with length of stay (p>0.05).

**Conclusion:**

There is a possibility of coronary artery patients to recover with normal blood pressure following reception of evidence-based cardiovascular nursing care.

## Introduction

Coronary artery disease (CAD) is the principal global cause of morbidity and mortality that is responsible for more economic costs than any other single illness 1,2. It is also the leading cause of death in China and increases economic health burden 3. According to the 2015 China Cardiovascular Diseases (CVD) report, an epidemic of CVD is emerging as a result of lifestyle changes, urbanisation and the accelerated process of aging 4. CAD is caused by atherosclerosis of the coronary arteries that leads to a restriction of blood flow to the heart^5^,^6^. The worse form of CAD known as acute coronary syndrome (ACS) contributes to emergencies of hospital admissions 2,7,8. Socio-demographic factors, such as old age, sex, family history of CAD and unhealthy lifestyles, obesity, smoking cigarettes and stress and comorbidities like hypertension, dyslipidaemia and diabetes mellitus have been universally reported as traditional risk factors of CAD 9–11.

Approximately 75% of CAD can be attributable to conventional risk factors 12. The modern treatment of CAD depends on the nature of coronary artery blockage or damage. These include vascular interventions such as percutaneous intervention (PCIs) (coronary stents and balloon angioplasty), coronary artery bypass graft (CABG), and pharmacological therapy 13,14. Patients have also benefited and improved their quality of life by attending cardiac rehabilitation programmes 15. As a result of this progress in treatment, the critical role of a nurse has also evolved.

## Nurses' roles and impact in caring for CAD patients

For more than four decades, nurses have been taking key roles in managing single and multiple risk factors of CAD. This has been done through specialised clinics and programmes in primary care, worksites, and cardiac rehabilitation centres 16,17. Provision of education on complex therapies in treating CAD, counselling on diet and lifestyle for risk factors modification and promotion on cardiac rehabilitation skills are among nursing roles delivered to patients with CAD 14. A systematic review of randomised controlled trials revealed that nursing interventions (NI) for CAD patients have a beneficial impact on blood pressure (BP), lipids, physical activity, dietary intake, cigarette smoking, weight loss, healthcare utilisation, mortality, quality of life, and psychosocial outcomes. However, more than half of the interventions (65%) were for education and behavioural counselling. Still inconsistent was the fact that many studies could not reveal which intervention feature was responsible for changes in outcome 18.

Another review of randomised controlled trials conducted in homes and outpatient settings determined statistically significant impact of coordinated cardiovascular nursing care (CVNC) on the reduction of BP to CAD patients 19.

The most recent systematic review and meta-analysis about the effective components of nurse-coordinated care to prevent recurrent coronary events also found heterogeneity of the data 20. However, authors used descriptive approach to summarise NI components and their effect on outcomes. Basing on their consensus, they distinguished three intervention strategies: (1) risk factor management, for example, prescription and or titration of drug therapy, education on risk factors, counselling on diet and life style modification, vital signs monitoring like BP and lipid control and stress/depressed mood detection and counselling;(2) multidisciplinary consultation, for example, consultation and referral and (3) shared decision making, for example, goal setting for individualised self-care plan and family support. Furthermore, important research questions were raised on how the proposed interventions can be translated into clinical practice, considering the fact that the statistically significant results may not represent clinically significant findings in all cases.

Basing on previous proposals, we published first the survey on the relevance of nursing caring interventions delivered to patients with CAD at a teaching hospital in Wuhan, China, by using patients' clinical records 21. Results basically found eight cardiovascular nursing caring interventions related to the recommended evidence that were delivered to CAD patients. We also found a high rate of hypertensive patients 224/300 (74.7%) with the history of about sixth readmission frequencies 165/300 (55%). This part of the study aroused further synthesis of our results from previous published data which showed that most patients were admitted with subnormal blood pressure (BP). Some school of thought nicknamed hypertension (HT) as truly a “silent killer” due to its significant association of contributing to acute myocardial infarction (AMI) by 4.8 times among hypertensive as compared to normal subjects 22. This implies a more indolent cardiac phenotype that if high BP is not well controlled would lead to more severe complications among CAD patients. Other studies suggested that preventive measures of HT at all levels should be given its special attention in minimising CAD episodes 23.

Taking into consideration that high BP was earlier reported as among the major risks of CAD, we thought it was important to understand if CAD patients admitted with subnormal BP got any improvement following their hospitalisation. Therefore, the purpose of the current study was to investigate the hypothesis which stated that “CVNC has an association with the (improvement of coronary artery patients' blood pressure) and LOS”. Publication of this study was expected to deliver further findings that appraise efforts of nurses in tackling CAD sequel to hospitalised patients and raise awareness on CAD illness to the readers of this article.

## Methods

### Study design and setting

This survey was based on cross-sectional retrospective design. This study was conducted using patient's clinical records from November 10, 2017, to September 18, 2018, in sub-acute CAD wards at a specialised large teaching hospital in Wuhan, China 21.

### Study participants and eligibility criteria

The study targeted hospital registry of discharged cardiovascular patients who were having CAD diagnosis within the specified period of time. CV patient case note was reviewed if it met the following criteria: (1) had CAD diagnosis (2) had complete records (paper, electronic, or both) (3) was 18 years and above and sex demographic information (4) had at least one CVNC documentation (5) ward management permitted to use patients' records for this study. Excluded files were those of below November 10, 2017, or if just CVD related files, collapsed/died cardiovascular case and or due to other unforeseen reasons.

### Sample size and sampling

A single population standard formula was used to calculate a sample size for this survey. This is a sample size calculated from a proportion of a single sampled study with similar or related characteristics of subjects for his study.

n= (Z) 2 p (1−p)/d^2^

n = Minimum sample size required for the study

Z = Value corresponding to the confidence level. For 0.05 confidence level = 1.96

p = Prevalence of stable CAD in previous studies taken as 22.8% 21

d = Absolute precision (tolerable error) = 0.05

n = (Z) 2 p (1−p)/d^2^

n = (1.96)2 0.23 (1–0.23)/ (0.05) ^2^

n = 0.68 = 272.12/0.0025

n = 272.12 + ((10%) n) (note: 10%–15% are standard percentages normally added for increasing the sample size)

n = 272.12 + 27.21 = 299.33

Then approximately 300 files were selected by simple random technique from 700 eligible CAD patients files through excel sheet system.

### Instrument and data collection

Data was collected by using a researcher-designed structured questionnaire. This approach was used due to the deficit of similar studies in the body of knowledge. Therefore, researchers designed the study questionnaire by adopting some categorical variables from previous related studies. However, “Any other (please specify)” option was purposively used to capture other information apart from those listed by the researchers in the questionnaire. The tool was divided into three sections. Section A: Composed of patient's demographic and clinical risk variables for CAD such as age, sex, smoking, and family history for CAD, hyperlipidaemia, diabetes mellitus and hypertension 10,11,24. Section B: Composed of CVNC for CAD patients; such as education and counselling (18); administration of CAD medication and their instructions such as antiplatelet, promotion on diet, lifestyle and risk modification, selfcare and cardiac rehabilitation 20,23,25,26. Part C: Composed of end clinical outcome variables such as length of stay (LOS) and discharge information of BP 27,28. Thereafter, the designed questionnaire was translated to meet local language needs. A panel of experts in the fields of nursing research, management, education and cardiovascular assessed the content validity of the questionnaire. Pretest of the tool was done in 15 (5%) questionnaires from clinical records of before November 2017. Also note that the pretesting did not include clinical records of died patients.

### Statistical analysis

Data was analysed by using the Statistical Package for Social Sciences Software (SPSS) version 21. Continuous variables were calculated as mean ± standard deviation (SD), and categorical variables were presented as counts (N) and percentages (%). Univariate and multivariate logistic regression models were used to compare the relationship of independent (CVNC) and dependent variables (BP and LOS). P-value of less than 0.05 and adjusted odds ratios (AOR) with 95% confidence interval (CI) within its ranges were stated as significant findings.

### Ethical considerations

The study protocol was approved by research ethics committee of Tongji Medical College of Huazhong University of Science and Technology (IORG#0003571). Furthermore, a verbal informed consent was obtained from hospitals' administrations to carry out the study and all patients details were kept anonymous.

## Results

Demographic and CAD risk comorbid characteristics of patients. We audited a total of 300 CAD patients' clinical records. Most were males 175(58.3%), their ages ranged from 33–99 with the mean age (63±11.2) years (Table 1). Majority were married, 267 (89%), while the rest were regarded as single and 278 (92.7%) used health insurance to access health care services. Their levels of education and income generation activities also varied diversely. Majority 224 (74.7%) were hypertensive. In these results, HT was among major modifiable CAD risk factors seen followed by diabetes mellitus 75 (25%) and hyperlipidaemia 46(15.3%). Other CAD risk life style behaviour like cigarette smoking was found to more than one third 126 (42.0%). About 105 (35.7%) of patients had CAD history in the family. Details of all demographics and most risky comorbidities of CAD patients are found in Table 1.

**Table 1:** Demographic and CAD risk comorbid characteristics of patients

Characteristics		n=300 (%)
Gender	Male	175 (58.3)
	Female	125 (41.7)
Age	Below mean age (63±11.2)	150 (50.0)
	From mean age (63±11.2) or above	150 (50.0)
Education level	High school or below	201(67.0)
	College or above	99 (33.0)
Marital status	aSingle	33 (11.0)
	Married	287 (89.0)
Admission frequency	First	135 (45.0)
	Second – sixth	165 (55.0)
Method of payment	Health insurance based	276 (92.0)
	Cash based	24 (8.0)
Occupation	Employed	73 (24.3)
	Retired or self-employed or student	227 (75.7)
Smoking	Yes	126 (42.0)
	No	174(58.0)
CAD in family	Yes	107 (35.7)
	No	193 (64.3)
Hypertension	Yes	224 (74.7)
	No	76 (25.3)
Hyperlipidaemia	Yes	46 (15.3)
	No	254 (84.7)
Diabetes Mellitus	Yes	75 (25.0)
	No	225 (75.0)

CAD, Coronary artery disease

a Single, includes widowed, separated and cohabiting

Comparing the association of CVNC with BP and LOS Tables 2 and 3 show statistical test results for comparing the association of cardiovascular nursing care with patients' blood pressure and length of stay. Note that documentation of this data was based on qualitative BP (whether normal or subnormal). Results show that less than quarter 40/300 (13.3%) of patients were discharged with subnormal BP (Table 1). Patients spent an average of eight days (7.7±1.6 days) of hospital admission. Categorical data analysis for LOS shows that about quarter 72/300 (24%) spent at least two weeks of hospitalisation (Table 2).

**Table 2:** Comparing the association of CVNC with stability of patients' blood pressure

Cardiovascular nursing care delivered	CVNC (n = 300 (%))		BP(n = 300) = N(260): SN(40)			

	Found	Not found	Crude OR (95%CI)	*p*-value	AOR(95%CI)	*p*-value
Assess to grade pain severity on 1–10 scale	206 (68.7)	94 (31.3)	2.4(1.1–5.6)	0.048*	3.1(1.2–7.8)	0.015*
Identify pain precipitating activities	210 (70.0)	90 (30)	2.2(0.9–5.2)	0.069	0.1(0.1–0.2)	0.999
Administer CAD medication and their instructions	290(96.7)	10 (3.3)	1.4(0.2–11.3)	0.754	0.1(0.1–0.2)	1.000
Educate on cardiac rehabilitation skills, exercise	285(95.0)	15 (5.0)	1.0(0.2–4.6)	1.000	1.5(0.1–17.9)	0.760
Counsel on diet and life style modification	289 (96.3)	11(3.7)	1.6(0.2–12.5)	0.676	4.7(0.4–5.6)	0.219
Offer reassurance and family counselling	284 (94.7)	16 (5.3)	0.4(0.1–1.4)	0.169	0.6(0.1–5.0)	0.678
Monitor vital signs	235 (78.3)	65 (21.7)	1.4(0.6–3.2)	0.493	1.3(0.4–3.7)	0.646
Counsel to cope with stress	60 (20.0)	240 (80.0)	4.8(2.4–9.8)	0.001**	5.8(2.8–12.2)	0.001**

CAD, Coronary artery disease; CVNC, Cardiovascular nursing care; BP, Blood pressure; N, Norma; SN, Sub-normal; OR, Odds Ratio; AOR, Adjusted Odds Ratio; CI, Confidence Interval

* p < 0.05

** p < 0.001

**Table 3:** Comparing the association of CVNC with patients' length of hospital stay

Cardiovascular nursing care	CVNC (n = 300 (%))		LOS(n =300) = 8D=228: 9–15D=72	
	
	Found	Not found	Crude OR (95%CI)	p-value
Assess to grade pain severity on 1–10 scale	206 (68.7)	94 (31.3)	1.1(0.6–1.9)	0.870
Identify pain precipitating activities	210 (70.0)	90 (30)	1.3(0.7–2.3)	0.444
Administer CAD medication and their instructions	290(96.7)	10 (3.3)	0.7(0.2–2.9)	0.653
Educate on cardiac rehabilitation skills, exercise	285(95.0)	15 (5.0)	0.4(0.2–1.3)	0.145
Counsel on diet and life style modification	289 (96.3)	11(3.7)	0.8(0.2–3.2)	0.796
Offer reassurance and family counselling	284 (94.7)	16 (5.3)	0.9(0.3–3.0)	0.923
Monitor vital signs	235 (78.3)	65 (21.7)	0.9(0.5–1.8)	0.896
Counsel to cope with stress	60 (20.0)	240 (80.0)	0.8(0.4–1.7)	0.636

CAD, Coronary artery disease; CVNC, Cardiovascular nursing care; LOS, Length of stay; D, Days; OR, Odds Ratio; CI, Confidence Interval

Both univariate and multivariate logistic regression analysis were run in SPSS 21 version to determine this association. In this analysis, CVNC was an independent variable on impacting patients' BP and LOS as dependent variables. After adjusting for patient's demographics, clinical characteristics and readmission information results show that CVNC, like “counsel patient to cope with stress” and “assess to grade pain severity on 1–10 scale,” were six and three times more likely to contribute to improved BP of patients following their hospitalisation (AOR=5.8; 95%CI: 2.8–12.2, p=0.001) and (AOR=3.1; 95%CI: 1.2–7.8, p=0.015) respectively (Table 2). However, no significant difference found with LOS (p >0.05) (Table 3).

## Discussion

The current study compared the association of cardiovascular nursing care (CVNC) with BP and LOS of hospitalised CAD patients at a large specialised teaching hospital in Wuhan, China. Mean age of patients was 63±11.2 years. This reflects older adults due to the fact that CAD is historically known as a disease of elderly as it develops gradually with aging. However, we observed half of the study population was younger than 60 years. This means CAD is currently affecting younger adults too. Our interpretation is similar to the finding which reported that CAD still affects majority of old adults with an upcoming trend of middle to younger adults^15^,^21^.

Consistently, the disease may become clinically apparent by age 40, but prevalence is higher in people 65 years of age and older 29.

From this study, three quarters of patients were hypertensive. This observation is similar to the findings where by hypertension had the leading proportion (80.7%) of all chronic comorbidity which were significantly associated with CAD in hospitalised cardiovascular patients^30^. In China, the prevalence of hypertension among subjects aged 15 years and above were reported to increase and also increase the risks of CVD and health expenditure. The major risk factors were high sodium and low potassium diet, obesity, overweight, high alcohol consumption, mental stress, family history of hypertension, and sedentary lifestyle 4. Several studies have listed hypertension as one of the leading comorbidities among CAD patients 31.

Our principle findings reveal significant association between CVNC “counsel patient to cope with stress” and “assess to grade pain severity on 1–10 scale” and BP. Where their relationship had positive beta values, hence indicates significant contribution of some nursing procedures in controlling patients from subnormal on admission to normal during discharge. The current study indicates that pain management has been one of the major focuses of nurses to CAD patients that had positive significant impact on BP. Nursing pain care is implemented as a response to a nursing diagnosis that is related by the bio physiologic injury secondary to decreased oxygen supply to the myocardium 32. Pain interventions offered go online and reflect the need of CAD patients whom cardiac chest pain is usually their major symptom. This finding is reflected in the results from a systematic review whereby nurse-led clinic group showed a significant decrease of worsening chest pain among patients who experienced angina as compared to those on usual care 19. Relieving chest pain would probably reflect improvement of coronary vascular blood flow, hence reduction of BP from vascular constriction.

However, fewer patients (20%) of 300 received nursing care on copying with stress.

Psychological factors such as depression and stress have been listed as among the underlying risk factors for CAD 33. Individuals who adjust CAD risk factors in a constructive direction can lower the risk for CAD^34^. Regardless of the fact that nurses can face time constraints and inadequate staffing, nursing practices can still be developed with technology that improves the physical environment to provide CAD patients with the opportunity to feel better about themselves 35. Therefore, we recommend increased efforts in managing patients' stress to minimise triggering CAD attacks for promoting patients' wellbeing.

This study faced difficulties of tracing some surgical and pharmacological records. The collaborative management was not adjusted for this study, hence should be of future focus to properly appraise the pure nursing efforts of relieving CAD patients' BP. Other studies revealed that multidisciplinary secondary prevention have a beneficial effect on process of care in patients with CAD 27. Therefore, future surveys can be done in similar or multiple settings to challenge these findings. Our findings are somehow consistent with other reports which revealed that the decline in-hospital acute CAD conditions like myocardial infarction (AMI) reflects advances in treatment and nurses efforts in timelines administration and monitoring side effects of reperfusion therapy^25^,^26^,^36^.

Moreover, the current findings show that patients spent almost a week with mean LOS (7.7±1.6) days. However, the current study has no enough evidence on the relationship of CVNC with LOS. Other findings reported that CAD patients with comorbidity conditions significantly spent more days of hospitalisation than those who had no chronic conditions 30. CAD patients were said to form a non-uniform group and their prognosis may differ depending on demographic, clinical, geographic, and socioeconomic factors 37. Therefore, basing on such findings, this relationship could probably be well explained by clinical characteristics or other factors.

Furthermore, we found more than half (55%) of 300 patients experienced and increased trend of readmissions from two to six times. This was also reported in studies where patients with CAD conditions like myocardial infarction (MI) faced some hospital readmission^27^.

Readmission frequencies could be associated with knowledge deficit in controlling their illness.

Studies explored that through knowledge assessment, patient education, discharge preparation, and care-coordination; nurses can be the frontline for providing many of the core processes of care aimed at preventing readmissions 38. Therefore, this study calls for a need of action in improving patients' knowledge of an illness and their well-being, hence minimise hospitalisation and related economic burdens to themselves, their families and nation at large.

To the best of our knowledge, availability of similar kind of study was limited, hence we faced deficit of enough comparable findings. However, benefits of CVNC that were found in the systematic reviews of randomised controlled trials have reflected current results 39. However, these kinds of studies are still inadequately conducted in the clinical settings. Hence further researches are needed to confirm its clinical significance.

Limitations of our survey include; being a retrospective of clinical patient's registry where some data was not complete, hence we couldn't get all numerical BP measurements of actual patients. Because of this, we opted for qualitative BP data (whether recorded as normal or subnormal), hence we were not able to measure differences of BP readings during hospital admission and discharge. This also limited the interview of patients who agreed to be discharged with unstable blood BP findings.

Additionally, there were insufficient surgical and pharmacological records as none nursing interventions to adjust in the statistical analysis of this association. Therefore, our study might have limited generalisability, hence could be appropriate for CVNC delivered to the population attending in the teaching hospital under this study.

## Conclusion

This study reveals that CAD is currently not only affecting older adults but also people below 60 years of age. Modifiable risk factors like hypertension are still increasing to compromise the health of CAD patients. Cardiovascular nursing care such as “counsel patient to cope with stress” and “assess to grade pain severity on 1–10 scale” significantly contributed to the correction of subnormal BP to normal of hospitalised CAD patients. However, this conclusion could be less solid due to small number of patients who received “help patient cope with stress” as well as insufficient comparison with other none nursing interventions. Hence further study is needed to fill these gaps and to properly appraise typical nursing efforts of caring CAD patients. Moreover, assessment of patients' knowledge on components on cardiac rehabilitation and related ill health behaviours is paramount in improving their health, minimising readmission frequencies and economic burden from CAD.
